# Bilateral sterile subdural effusion in Kawasaki disease-A case report

**DOI:** 10.3389/fped.2022.990544

**Published:** 2022-09-12

**Authors:** Corina Ramona Nicolescu, Marie Duperril, Jean-Louis Stephan

**Affiliations:** ^1^Pediatric Department, University Hospital Saint-Etienne, Saint-Etienne, France; ^2^Pediatric Intensive Care Unit, University Hospital Saint-Etienne, Saint-Etienne, France

**Keywords:** Kawasaki disease, medium vessel vasculitis, inflammation, aseptic meningitis, subdural collection

## Abstract

Kawasaki disease is an acute febrile condition that causes a self-limiting medium vessel systemic vasculitis and whose pathophysiological pathways are still not completely understood. Coronary arteries are the most affected, but inflammation can develop in all medium-sized arteries, with various organs and tissues being involved. Kawasaki disease-related neurological involvement varies in terms of clinical expression and severity. Herein, we describe an unusual neurological complication of Kawasaki disease in a 5-year-old girl. The progression of the disease was biphasic. Kawasaki disease was diagnosed on the 8th day after symptoms onset and treated by intravenous immunoglobulins, with prompt clinical regression but a less favorable biological response (persistent inflammation with hypoalbuminemia). Two weeks later, headaches and lethargy developed, and a bilateral subdural collection was identified on cerebral imaging. Subsequently, her progress was uneventful, with no residual coronary abnormalities and complete resorption of the subdural collection. Bilateral subdural collection, exceptionally reported, could be discussed as a clinical expression of systemic inflammatory vasculitis that characterizes Kawasaki disease.

## Introduction

Kawasaki disease (KD) is an acute febrile illness of childhood characterized by clinical, laboratory, and histopathological features of medium-sized systemic vasculitis, with a significant predilection for the coronary arteries.

Its etiopathogenesis is still not fully understood and appears to be an interplay of several dysregulated molecular mechanisms, including genetic susceptibility and microbe-derived innate immune responses. Vasculitis involvement in KD could be interpreted as an unusual immune response (genetically determined) to a common environmental stimulus (1).

As systemic vasculitis, inflammation can develop in all medium-sized arteries and in various organs and tissues during the acute febrile phase (2), with variable clinical features (cardiac, hepatic, pulmonary, gastrointestinal, renal, neurological, or muscular) (3).

KD classical clinical picture mirrors the widespread vascular inflammation and develops as febrile illness (5 days or more) with characteristic bilateral bulbar non exudative conjunctivitis, oral mucous membrane erythema, biphasic peripheral extremity changes, (erythema of palms or soles, edema of hands or feet during the acute phase and periungual desquamation during convalescent phase), polymorphous rash and cervical lymphadenopathy (at least 1 lymph node >1.5 cm in diameter) (4). Irritability with unexplained behavioral changes are frequently observed, particularly in young children.

An incomplete KD is diagnosed when the classical criteria are not fulfilled (5). Young children are febrile for 5 days or more, present with few clinical features, and laboratory evidence of systemic inflammation. The diagnosis is challenging, and the echocardiography performed early in the evaluation allows correct diagnosis and timely treatment.

Neurological involvement during KD progression is variable in terms of clinical features/diagnosis and severity, such as irritability, facial nerve palsy, sensorineural hearing loss, ataxia, seizures, aseptic meningitis, encephalitis, cerebral infarction, and subdural effusion. Subdural effusion is infrequent, with only several cases reported in infants.

Herein, we report a new clinical observation of KD complicated by aseptic meningitis and extensive bilateral subdural collection in a 5-year-old girl.

## Case report

A previously healthy 5-year-old girl presented to the Emergency Department (ED) with high-spiking fever (39–40°C) lasting for 6 days, asthenia, and minor respiratory symptoms. Her medical history and family history were unremarkable.

At admission, she looked well and non-toxic. Her vital signs were notable for a temperature of 40°C and tachycardia. Findings from physical examination, including neurological evaluation, were normal. She presented no clinical features for KD diagnosis. Shortly after arrival in the ED, she presented an episode of focal, secondarily generalized, tonic-clonic seizures that responded to benzodiazepines (intravenous midazolam and clonazepam).

Initial laboratory evaluation showed severe systemic inflammation: neutrophilic leucocytosis (white blood count, WBC 19,800/mL with 73% neutrophils), elevated C-reactive protein (CRP) (377 mg/L), procalcitonin (13.68 μg/L), ferritin (738 μg/L), fibrinogen (9.8 g/L), D-dimers (4000 ng/mL) levels and hypoalbuminemia (3.2 g/dL). Her complete biological panel was normal, except thrombocytosis (platelet count 574,000/mm^3^), normocytic normochromic anemia (hemoglobin 9.5 g/dL), mild hyponatremia (129 mmol/L), and sterile pyuria.

Lumbar puncture showed a clear cerebrospinal fluid (CSF), and CSF analysis revealed a moderately increased cell count (WBC 25/mm^3^, neutrophils 73%, no erythrocytes) and protein content (0.54 g/L) as well as normal glucose (4.43 mmol/L) and lactate (2.17 mmol/L). The meningitis/encephalitis panel polymerase chain reaction (PCR) spinal fluid results were negative. The nasopharyngeal aspirate for respiratory viral panel yielded negative results. Epstein-Barr virus, cytomegalovirus, parvovirus, enterovirus and adenovirus infection were excluded by PCR testing. The results of blood, CSF, and urine bacterial cultures were negative. 16SrDNA sequencing of bacteria in CSF was also negative. The IgG antibodies to the SARS CoV-2 virus were negative and she had no exposure history.

Echocardiogram showed dilated coronary arteries, minimal pericardial effusion, and normal ventricular contractility. The diameters of the dilated vessels were as follows: left anterior descending artery (LADA) 2.8 mm (+ 2 DS), and right coronary artery (RCA) 2.7 mm (+ 2DS). Additional assessments for cardiac involvement demonstrated no ECG changes and normal troponin levels.

Incomplete KD was diagnosed and treatment with high-dose intravenous immunoglobulins (IVIG) 2 g/kg and acetylsalicylic acid (75 mg/kg/day) was started on the 8th day after fever onset.

Fever defervescence was obtained over 24 h, with a favorable clinical progress (no new neurological symptoms or systemic KD signs), but an unchanged inflammatory response. Steroids (intravenous methylprednisolone) were added in an attempt to reduce the inflammation, and she was discharged on the 15th day after disease onset, on acetylsalicylic acid 75 mg/kg/day and prednisolone 2 mg/kg/day.

On day 22 of symptom onset, while at home, she experienced severe headaches, became lethargic, and was readmitted. Upon examination, she was in pain, and had normal vital signs as well as a strictly normal physical exam.

**Figure 1:**
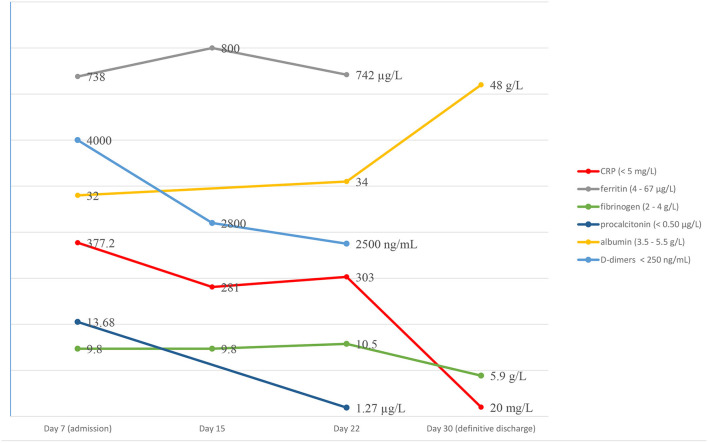
Biological course.

Laboratory examination found a persistent inflammatory state with neutrophilic leukocytosis (WBC 35,000/mm^3^, neutrophils 80%) and CRP at 303 mg/L (Figure 1). A second lumbar puncture was performed, which showed an inflammatory fluid with increased pleocytosis (394 white blood cells/mm^3^, neutrophils 74%) and normal biochemistry (normal glucose and protein content).

**Figure 2:**
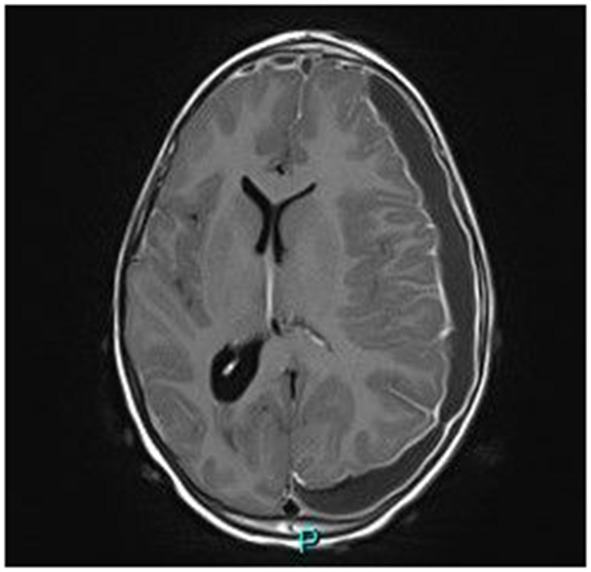
Axial gadolinium-enhanced T1 MR Image—bilateral hyper intense subdural fluid collection surrounded by a contrast-enhancing rim. (larger on the left side) with left-to-right midline shift. Neither parenchymal involvement nor related cerebral oedema.

Magnetic resonance imaging (MRI) demonstrated a large bilateral front-temporal subdural collection (much more extensive to the left side) with a significant mass effect. No parenchymal intensity areas were detected (Figure 2).

Neurosurgical drainage was performed; macroscopically, the CSF had a pus-like appearance, and microscopically, it was very inflammatory, with predominance of neutrophils. No bacteria were identified on 16s rDNA sequence analysis of the subdural collection fluid. No common source (neurosurgical procedure, skull trauma, otogenic, or mastoid sources) to explain the possible infectious origin of this collection was identified.

The radiological characteristics of cerebral collection and the biological aspect of CSF/collection fluid were consistent with the diagnosis of subdural collection, most probably of inflammatory origin. Despite the lack of any viral and bacterial illnesses, empirical antibiotic (amoxicillin/clavulanic acid) treatment was started.

Echocardiographic monitoring (on the 28th day of disease) revealed that the coronary artery abnormalities/dilatation had subsided, pericardial effusion had resorbed, and no additional morphological or functional damage was identified.

On the 31st day of disease, the child was afebrile, with a normal clinical/neurological examination and considerably diminished inflammatory condition (CRP 32 mg/L, fibrinogen 4 g/L, albumin 4.8 g/dL). She was discharged and the follow-up (clinical and imagistic) documented normal results in the following 12 months.

**Figure 3:**
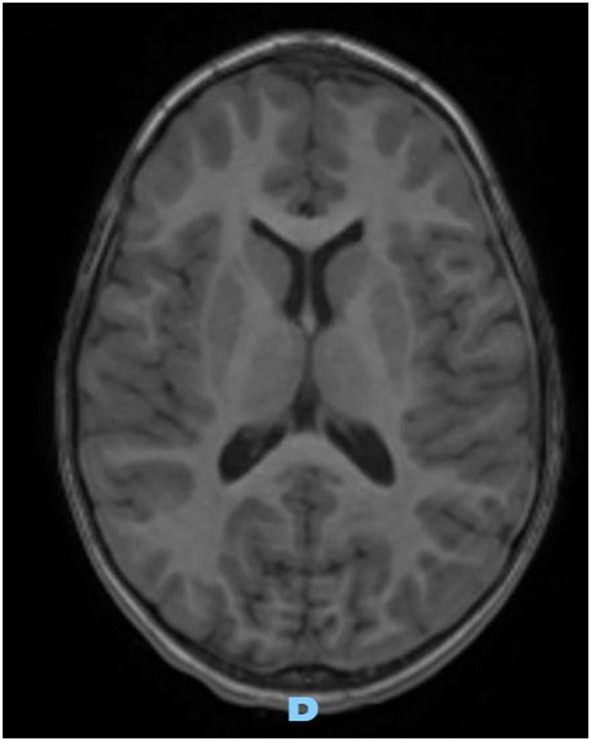
Axial gadolinium-enhanced T1 MR Image—spontaneous and complete resolution of subdural collection.

On the first follow-up brain MRI (1 month later), the left collection was significantly smaller, and the right one resolved completely, without complication. Three months later, follow-up MRI images were normal (Figure 3).

## Discussion

Our patient presented an incomplete KD with cardiac (coronary dilation, minimal pericardial effusion) and neurological involvement (seizures, aseptic meningitis, and subdural collection) in the context of a marked and prolonged inflammation and negative comprehensive infectious screening.

In April 2020, a new multisystem inflammatory entity affecting children in relation to SARS-CoV-2 infection termed multisystem inflammatory syndrome in children (MIS-C) was reported. Its diagnosis requires clinical (persistent fever, oral mucosal and conjunctiva changes, digestive, respiratory symptoms, myocardial, and left ventricular dysfunction of variable severity), biological (hyper inflammation) features in the context of confirmed SARS-CoV-2 infection or exposure (6).

Although many patients could meet both KD and MIS-C criteria, suggesting that both entities are due to viral-induced inflammatory response secondary to cytokine system activation, several clinical, biological, therapeutic response distinctions remain, differentiating KD and MIS-C.

In our patient, MIS-C was discussed as differential diagnosis, but her age, and all clinical, biochemical and imaging data did not support this diagnosis.

Neurological manifestations have been reported in association with KD (7), which are currently well known, appearing in 1–30% of cases (3). Their clinical expression and severity are diverse, and the most frequently described neurological involvements include seizure, facial nerve palsy, meningoencephalitis, mild encephalopathy with reversible splenial lesions, hemiplegia, ataxia, chorea, ischemia, abnormal vision, disturbed consciousness, behavioral abnormalities, sensorineural hearing loss, and monocyte-predominant pleocytosis in CSF (8, 9).

In a recent retrospective study (10) that recorded neurological involvements in 1,582 patients diagnosed with KD, the incidence of such complications was low (5.1%) and associated with a more severe inflammatory response and reduced efficacy of IVIG therapy.

No significant association between coronary artery abnormalities and neurological involvement was found.

Subdural collection remains a rare complication of KD. In 1990, six cases were reported in Japanese patients (11, 12), and since then, new cases have been reported, which are summarized in Table 1.

**Table 1:** Clinical presentations of patients who had subdural collection associated with Kawasaki disease.

**Age at diagnosis**	**Diagnosis**	**Neurologic complication**	**Treatment**	**Follow-up**	**Reference**
4-month-10-day-old boy	KD No coronary abnormalities	Bilateral subdural effusion	IVIG aspirin	2 months Brain US normal 12 months Normal development	(18)
5-month-old girl	KD Mild focal coronary dilatation	Fluid collection in the epidural space	IVIG aspirin	Normal brain MRI Normal development	(19)
6-month-old boy	KD No coronary abnormalities	Bilateral subdural collection	IVIG aspirin	6 months CT brain normal Normal development	(13)
A group of 5 Japanese patients Age between 3 and 15 months	KD	Fluid collection in the frontal extra cerebral space monocyte-predominant pleocytosis			(12)
6-month-old girl	KD	Bilateral subdural fluid collection Intracranial hypertension Retinal hemorrhage . Aseptic meningitis	Subdural tap	6 months Normal development CT brain Definite reduction in the volume of the subdural effusion	(11)

KD, Kawasaki disease; IVIG, intravenous immunoglobulines; US, ultrasound; CT, computer tomography.

Several common features could be retained from these published cases: 1) the young age of the patients, 2) neurological involvement expressed clinically during the acute phase of KD, 3) laboratory biomarkers indicating severe inflammation and in one case (12) hypoalbuminemia, 4) all patients except one had bilateral collection, and they did not undergo surgical evacuation or antibiotic therapy, and 5) the neurologic prognosis (clinical and radiological) was favorable, with complete recovery in all cases (except one child who developed mild hearing impairment) (13).

The postulated pathophysiologic mechanism of the subdural effusion is related to intracranial inflammation (CSF pleocytosis) and inflammatory vasculitis of the dura mater.

Inflammatory pathway was also proposed to explain hypoalbuminemia. Plasma albumin is a negative acute-phase reactant, and inflammation induces an increased vascular permeability (leaky vessels) (14, 15).

The patient in this case report presented an initial brief episode of complex seizures on admission, in the context of aseptic meningitis. This initial neurological picture might be part of the systemic inflammatory disease in keeping with the raised systemic inflammatory markers.

Febrile seizures in the acute phase of KD are very rare. A retrospective analysis of the incidence of this neurological manifestation in patients with KD found only two patients (of 177), who presented generalized seizures associated with prolonged consciousness disturbance and pleocytosis in the CSF (16). No mechanism/hypothesis has been discussed to explain the absence of paroxysmal neurological manifestations in children with KD and cerebral vasculitis confirmed by CSF biochemistry abnormalities.

During the remission phase of the disease, our patient presented afebrile headaches and lethargy, and the diagnosis of bilateral asymmetrical subdural collection was radiologically made, which was considered to be inflammatory origin (severe and persistent inflammatory syndrome, with no evidence of infection on PCR or culture). Dura mater involvement in KD is well-known, although it remains an event with variable clinical expression during the disease.

Our case raised several questions: a) is it reasonable to assume that the initial aseptic meningitis and the consecutive development of the extra cerebral collection are part of the same inflammatory process involving the dura mater? b) could we associate these two complications to the severity and persistence of the inflammatory changes (inflammatory syndrome, hypoalbuminemia, leucocytosis and CSF pleocytosis) over 4 weeks? c) could neurological involvement be a marker of disease severity or inflammation? d) could an immunomodulatory treatment, including interleukin-targeted therapy or corticosteroids be more appropriate than conventional treatment with immunoglobulins alone in case of severe and prolonged inflammatory response? Some answers are available in the recent literature.

A bilateral subdural collection of inflammatory origin (vasculitis) in a patient with neuro-Behcet disease resolved under steroid pulsed therapy (16). A recent report (17) of 4 patients with central (acute splenial lesions) and peripheral (global proximal muscle weakness and reduced reflexes) neurological involvement in the context of COVID-19 MIS-C describes a favorable neurological outcome with immunomodulatory therapies as intravenous methylprednisolone, dexamethasone, intravenous immunoglobulin anakinra, and rituximab.

## Conclusion

We suggest that cerebral involvement in KD, particularly subdural effusion may not be accidental. From a clinical and pathophysiological perspective, its early diagnosis is challenging and could improve our global comprehension of inflammatory mechanisms, with cytokine storm and immune system activation in KD, avoid invasive studies and allow early use of potentially effective second-line therapy like immune-modulatory agents/interleukin blockers.

## Data availability statement

The raw data supporting the conclusions of this article will be made available by the authors, without undue reservation.

## Author contributions

All authors listed have made a substantial, direct, and intellectual contribution to the work and approved it for publication.

## Conflict of interest

The authors declare that the research was conducted in the absence of any commercial or financial relationships that could be construed as a potential conflict of interest.

## Publisher's note

All claims expressed in this article are solely those of the authors and do not necessarily represent those of their affiliated organizations, or those of the publisher, the editors and the reviewers. Any product that may be evaluated in this article, or claim that may be made by its manufacturer, is not guaranteed or endorsed by the publisher.

## References

[1] Rivas MN Arditi M. Kawasaki disease: pathophysiology and insights from mouse models. Nat Rev | Rheumatol. (2020) 16:391. 10.1038/s41584-020-0426-0 32457494 PMC7250272

[2] Amano S Hazama F Kubagawa H Tasaka K Haebara H Hamashima Y. General pathology of Kawasaki disease: on the morphological alterations corresponding to the clinical manifestations. Acta Pathol Jpn. (1980) 30:681–94. 10.1111/j.1440-1827.1980.tb00966.x 7446109

[3] Tizard EJ. Complications of Kawasaki disease. Curr Pediatr. (2005) 15:62–8. 10.1016/j.cupe.2004.09.002

[4] Newburger JW Takahashi M Gerber MA Gewitz MH Tani LY Burns JC. Diagnosis, treatment, and long-term management of Kawasaki disease: a statement for health professionals from the Committee on Rheumatic Fever, Endocarditis, and Kawasaki Disease, Council on Cardiovascular Disease in the Young, American Heart Association. Pediatrics. (2004) 114:1708–33. 10.1542/peds.2004-2182 15574639

[5] McCrindle BW Rowley AH Newburger JW Burns JC Bolger AF Gewitz M. Diagnosis, treatment, and long-term management of Kawasaki disease: a scientific statement for health professionals from the American heart association. Circulation. (2017) 135:e927–99. 10.1161/CIR.0000000000000484 28356445

[6] Bar-Meir M Guri A Godfrey ME„ Shack AR Hashkes PJ Goldzweig O Megged O. Characterizing the differences between multisystem inflammatory syndrome in children and Kawasaki disease. Sci Rep. (2021) 11:13840 10.1038/s41598-021-93389-0 34226639 PMC8257717

[7] Terasawa K Ichinose E Matsuishi T Kato H. Neurological complications in Kawasaki Disease. Brain Dev. (1983) 5:371–4. 10.1016/S0387-7604(83)80041-2 6638393

[8] Tabarki B Mahdhaoui A Selmi H Yacoub M Essoussi AS. Kawasaki disease with predominant central nervous system involvement. Pediatr Neurol. (2001) 25:239–41. 10.1016/S0887-8994(01)00290-9 11587880

[9] Kontzialis M Soares BP Huisman T. Lesions in the splenium of the corpus callosum on mri in children: a review. J Neuroimaging. (2017) 27:549–61. 10.1111/jon.12455 28654166

[10] Liu X Zhou K Hua Y Wu M Liu L Shao S. Neurological involvement in Kawasaki disease: a retrospective study. Paediatr Rheumatol. (2020) 18:61. 10.1186/s12969-020-00452-7 32664982 PMC7362431

[11] Aoki N. Subdural effusion in the acute stage of Kawasaki disease (Mucocutaneous lymph node syndrome). Surg Neurol. (1988) 29:216–7. 10.1016/0090-3019(88)90009-2 3344468

[12] Takagi K Umezawa T Saji T et al. Meningoencephalitis in Kawasaki disease. No To Hattatsu. (1990) 22:429–35. 2223179

[13] Bailie NM Hensey OJ Ryan S Allcut D King MD. Bilateral subdural collections–an unusual feature of possible Kawasaki disease. Eur J Pediatr Neurol. (2001) 5:79–81. 10.1053/ejpn.2001.0469 11589317

[14] Suzuki N Takeno M Inaba G. Bilateral subdural effusion in a patient with neuro-Behçet's disease. Ann Rheum Dis. (2003) 62:374–37. 10.1136/ard.62.4.374 12634247 PMC1754514

[15] Ronit A Kirkegaard-Klitbo DM Dohlmann TL Lundgren J Sabin CA Phillips AN et al. Plasma albumin and incident cardiovascular disease. Arterioscler Thromb Vasc Biol. (2020) 40:473–82. 10.1161/ATVBAHA.119.313681 31852221

[16] Yoshkawa H Abe T. Febrile convulsion during the acute phase of Kawasaki disease. Paediatr Int. (2004) 46:31–2. 10.1111/j.1442-200X.2004.01850.x 15043661

[17] Abdel-Mannan O Eyre M Lobel U Bamford A Eltze C Hameed B. Neurologic and radiographic findings associated with COVID-19 infection in children. JAMA Neurol. (2020) 77:1440–5. 10.1001/jamaneurol.2020.2687 32609336 PMC7330822

[18] Chou CPI Lin C Kuo KC. A male infant had subdural effusion and paroxysmal supraventricular tachycardia during the febrile episode of Kawasaki disease: a case report and literature review. BMC Pediatr. (2016) 16:71. 10.1186/s12887-016-0606-x 27234442 PMC4884381

[19] Jung-OK K Lee HJ Han KH. Incomplete Kawasaki disease in a 5-month-old girl associated with cereborspinal fluid pleocytosis and epidural fluid collection. Pediatr Infect Vaccine. (2015) 22:40–4. 10.14776/piv.2015.22.1.40

